# Supplementary material for: Experimental evidence that penis size, height, and body shape influence assessment of male sexual attractiveness and fighting ability in humans
Source: PLoS Biol. 2026 Jan 22;24(1):e3003595. doi: 10.1371/journal.pbio.3003595 (PMC12826512; doi:10.1371/journal.pbio.3003595)
Supplement: S1 Text — Table A in S1 Text. Final sample size per survey. For each survey, data were collected from >100 participants, but some participants were excluded (see Materials and methods). Table B in S1 Text. Outputs from likelihood ratio tests assessing differences in the selection matrix due to mode of delivery, participant sex, and assessment factor (attractiveness vs. fighting ability). Bold values indicate statistical significance after FDR correction. Table C in S1 Text. Linear (β) and quadratic/correlational (γ) selection gradients based on means of gradients generated for each participant for in-person (A) and online (B) surveys. Asterisks indicate significance after FDR correction. Table D in S1 Text. General linear mixed model results for all response time as the dependent variable and the three standardized male traits as fixed covariates for in-person and online surveys. Bold values indicate statistical significance after FDR correction. (DOCX) [file pbio.3003595.s001.docx]

**S1 Text. Supplementary tables (A-D).**

**Experimental evidence that penis size, height and body shape influence assessment of male sexual attractiveness and fighting ability in humans**

**Table A**. Final sample size per survey. For each survey, data was collected from >100 participants, but we then had to exclude some participants (see Materials and Methods).

| Sex of  participant | Assessment | In-person | Online (unpaid) | Online (paid) |
| --- | --- | --- | --- | --- |
| Male | Fighting ability | 100 | 97 | 85 |
| Male | Attractiveness | 102 | 90 | 92 |
| Female | Attractiveness | 105 * | 94 | 90 |

*data from Mautz et al., (2013) [1]

**Table B.** Outputs from the **likelihood ratio tests** that we used to test for differences in the selection matrix due to the mode of delivery (in-person or online), sex of the participant, and whether the participant was rating attractiveness or fighting ability. Bold values indicate statistical significance with original *P* values after accounting for a false discovery rate for multiple comparisons (see Methods).

| Did mode of delivery (in-person vs online) affect ranking? | | |  |  |  |
| --- | --- | --- | --- | --- | --- |
| **Mode** |  | **Sum of sq** | **Df** | **F** | ***P (F)*** |
| In-person vs online unpaid | Female rating of male attractiveness | -31.288 | -10 | 30.463 | **< 0.000001** |
|  | Male rating of rival’s attractiveness | -9.2621 | -10 | 20.678 | **< 0.000001** |
|  | Male rating of rival’s fighting ability | -14.461 | -10 | 37.961 | **< 0.000001** |
|  |  |  |  |  |  |
| Online paid vs unpaid | Female rating of male attractiveness | -3.1503 | -10 | 3.1292 | **0.0006** |
|  | Male rating of rival’s attractiveness | -7.8651 | -10 | 17.693 | **< 0.000001** |
|  | Male rating of rival’s fighting ability | -5.418 | -10 | 9.606 | **< 0.000001** |
|  |  |  |  |  |  |
| In-person vs online paid | Female rating of male attractiveness | -48.377 | -10 | 41.549 | **< 0.000001** |
|  | Male rating of rival’s attractiveness | -5.6256 | -10 | 10.823 | **< 0.000001** |
|  | Male rating of rival’s fighting ability | -13.443 | -10 | 21.215 | **< 0.000001** |
| Did participant sex and assessment factor (attractiveness vs fighting) affect ranking? | | | | | |
| **Sex and assessment factor** | | **Sum of sq** | **Df** | **F** | ***P (F)*** |
| In-person | Male vs female rating of attractiveness | -58.703 | -10 | 68.847 | **< 0.000001** |
|  | Male rating of fight ability vs attractiveness | -27.506 | -10 | 56.517 | **< 0.000001** |
|  | Female attractiveness vs male fighting | -104.89 | -10 | 128.49 | **< 0.000001** |
|  |  |  |  |  |  |
| Online unpaid | Male vs female rating of attractiveness | -52.846 | -10 | 85.025 | **< 0.000001** |
|  | Male rating of fight ability vs attractiveness | -6.6487 | -10 | 19.432 | **< 0.000001** |
|  | Female rating of attractiveness vs male rating of fighting ability | -76.775 | -10 | 129.97 | **< 0.000001** |
|  |  |  |  |  |  |
| Online paid | Male vs female rating of attractiveness | -24.405 | -10 | 29.401 | **< 0.000001** |
|  | Male rating of fight ability vs attractiveness | -15.967 | -10 | 23.948 | **< 0.000001** |
|  | Female rating of attractiveness vs male rating of fighting ability | -57.052 | -10 | 58.211 | **< 0.000001** |

**Table C.** Linear selection gradients (β) and the matrix (γ) of quadratic (on diagonal) and correlational (below diagonal) selection gradients based on means of gradients generated separately for each participant for **A** in-person and **B** online surveys. *P* values are from one-sample *t*-tests for a mean value of zero. Bold values indicate statistical significance. Asterisks indicate FDR rate at ****P* < 0.001, ***P* < 0.01, **P* < 0.05 (see Methods).

| **Female rating of male attractiveness** | | | | | | | | |
| --- | --- | --- | --- | --- | --- | --- | --- | --- |
|  |  |  | Quadratic (γ) | | | | | |
| Trait | Linear (β) | | Penis size | | Height | | Body shape | |
|  | A | B | A | B | A | B | A | B |
| Penis size | **0.249***** | **0.511***** | **-0.063**** | **-0.087***** |  |  |  |  |
| Height | **0.279***** | **0.126***** | **0.043**** | 0.027 | **-0.057**** | -0.027 |  |  |
| Body shape | **1.078***** | **0.760***** | **0.064**** | **0.116***** | **0.090***** | **0.073***** | **-0.145***** | **-0.111***** |
| **Male rating of rival’s attractiveness** | | | | | | | | |
|  |  |  | Quadratic (γ) | | | | | |
| Trait | Linear (β) | | Penis size | | Height | | Body shape | |
|  | A | B | A | B | A | B | A | B |
| Penis size | **0.294***** | **0.215***** | 0.015 | **0.028**** |  |  |  |  |
| Height | **0.209***** | **0.057**** | 0.006 | 0.012 | 0.028 | 0.011 |  |  |
| Body shape | **0.470***** | **0.315***** | **0.043*** | **0.037**** | **0.072***** | 0.026 | -0.014 | -0.012 |
| **Male rating of rival’s fighting ability** | | | | | | | | |
|  |  |  | Quadratic (γ) | | | | | |
| Trait | Linear (β) | | Penis size | | Height | | Body shape | |
|  | A | B | A | B | A | B | A | B |
| Penis size | **0.057*** | **0.079**** | 0.014 | 0.008 |  |  |  |  |
| Height | **0.500***** | **0.188***** | 0.009 | -0.008 | **0.045**** | 0.008 |  |  |
| Body shape | **0.313***** | **0.298***** | -0.007 | **0.030**** | **0.042**** | 0.03 | 0.003 | 0.011 |
|  |  |  |  |  |  |  |  |  |

**Table D.** Results from general linear mixed models with parameter estimates and chi-square (χ2) test statistics, including all **response time** as the dependent variable and the three standardised male traits as fixed covariates for: a) female rating of male attractiveness; b) male rating of rival’s attractiveness; and c) male rating of rival’s fighting ability for in-person and online surveys. Bold values indicate statistical significance with original *P* values after accounting for a false discovery rate for multiple comparisons (see Methods).

| ***1. In-person*** | |  |  | ***2. Online*** | |  |  |
| --- | --- | --- | --- | --- | --- | --- | --- |
| **a. Female rating of male attractiveness** | | | | | | | |
| Traits | Estimate | χ2 | *P* | Traits | Estimate | χ2 | *P* |
| (Intercept) | 7.869 |  |  | (Intercept) | 8.595 |  |  |
| Penis size | 0.027 | 15.431 | **0.00009** | Penis size | 0.042 | 26.2 | **<0.00001** |
| Height | 0.024 | 12.452 | **0.0004** | Height | 0.02 | 5.891 | **0.015** |
| Body shape | 0.11 | 257.851 | **<0.00001** | Body shape | 0.05 | 37.236 | **<0.00001** |
| **b. Male rating of rival’s attractiveness** | | | | | | | |
| Traits | Estimate | χ2 | *P* | Traits | Estimate | χ2 | *P* |
| (Intercept) | 7.843 |  |  | (Intercept) | 8.117 |  |  |
| Penis size | 0.049 | 34.052 | **<0.00001** | Penis size | 0.027 | 8.582 | **0.003** |
| Height | 0.032 | 14.105 | **0.0002** | Height | 0.019 | 4.418 | 0.036 |
| Body shape | 0.083 | 98.138 | **<0.00001** | Body shape | 0.05 | 29.691 | **<0.00001** |
| **c. Male rating of rival’s fighting ability** | | | | | | | |
| Traits | Estimate | χ2 | *P* | Traits | Estimate | χ2 | *P* |
| (Intercept) | 7.562 |  |  | (Intercept) | 8.213 |  |  |
| Penis size | 0.001 | 0.007 | 0.932 | Penis size | 0.028 | 9.6 | **0.002** |
| Height | 0.049 | 38.861 | **<0.00001** | Height | 0.017 | 3.555 | 0.059 |
| Body shape | 0.024 | 9.225 | **0.002** | Body shape | 0.043 | 22.707 | **<0.00001** |

**Reference**

1. Mautz, B. S., Wong, B. B., Peters, R. A., & Jennions, M. D. (2013). Penis size interacts with body shape and height to influence male attractiveness. *Proceedings of the National Academy of Sciences*, *110*(17), 6925-6930.
