# Supplementary material for: Experimental evidence that penis size, height, and body shape influence assessment of male sexual attractiveness and fighting ability in humans
Source: PLoS Biol. 2026 Jan 22;24(1):e3003595. doi: 10.1371/journal.pbio.3003595 (PMC12826512; doi:10.1371/journal.pbio.3003595)
Supplement: S1 Appendix — (DOCX) [file pbio.3003595.s002.docx]

**Experimental evidence that penis size, height and body shape influence assessment of male sexual attractiveness and fighting ability in humans**

**APPENDIX**

**Analyses of all paid online data**

**Table A**. Linear selection gradients (β) and the matrix (γ) of quadratic (on diagonal) and correlational (below diagonal) selection gradients based on the average rating for each of the 343 figures (i.e., no control for multiple participant responses) for **paid online surveys**. Bold values indicate statistical significance. Asterisks indicate FDR rate all paid data at ****P* < 0.001, ***P* < 0.01, **P* < 0.05 (see Methods).

| **Female attractiveness** | | | | |
| --- | --- | --- | --- | --- |
|  | Linear (β) | Quadratic (γ) | | |
| Trait |  | Penis size | Height | Body shape |
| Penis size | **0.602***** | **-0.096*** |  |  |
| Height | **0.105***** | **0.05**** | -0.04 |  |
| Body shape | **0.713***** | **0.107***** | **0.038*** | **-0.346***** |
| **Male rating of rival’s attractiveness** | | | | |
|  | Linear (β) | Quadratic (γ) | | |
| Trait |  | Penis size | Height | Body shape |
| Penis size | **0.391***** | **0.144***** |  |  |
| Height | **0.082***** | -0.006 | 0.006 |  |
| Body shape | **0.415***** | **0.056***** | 0.021 | **-0.084**** |
| **Male rating of rival’s fighting ability** | | | | |
|  | Linear (β) | Quadratic (γ) | | |
| Trait |  | Penis size | Height | Body shape |
| Penis size | **0.144***** | **0.14***** |  |  |
| Height | **0.251***** | -0.029 | 0.028 |  |
| Body shape | **0.426***** | 0.004 | **0.037*** | **-0.11**** |

**Table B.** Linear selection gradients (β) and the matrix (γ) of quadratic (on diagonal) and correlational (below diagonal) selection gradients based on means of gradients generated separately for each participant for **paid online surveys**. P values are from *t*-tests for a significant difference between the mean gradient and zero. Bold values indicate statistical significance. Asterisks indicate FDR rate for all paid data ****P* < 0.001, ***P* < 0.01, **P* < 0.05 (see Methods).

| **Female rating of male attractiveness** | | | | |
| --- | --- | --- | --- | --- |
|  |  | **Quadratic (γ)** | | |
| Trait | Linear (β) | Penis size | Height | Body shape |
| Penis size | **0.640***** | -0.007 |  |  |
| Height | 0.072 | 0.035 | -0.029 |  |
| Body shape | **0.723***** | **0.113***** | 0.034 | **-0.148***** |
| **Male rating of rival’s attractiveness** | | | | |
|  |  | **Quadratic (γ)** | | |
| Trait | Linear (β) | Penis size | Height | Body shape |
| Penis size | **0.402***** | **0.072***** |  |  |
| Height | **0.076**** | -0.011 | 0.018 |  |
| Body shape | **0.422***** | **0.056***** | 0.018 | **-0.042**** |
| **Male rating of rival’s fighting ability** | | | | |
|  |  | **Quadratic (γ)** | | |
| Trait | Linear (β) | Penis size | Height | Body shape |
| Penis size | **0.151***** | 0.044 |  |  |
| Height | **0.278***** | -0.025 | 0.015 |  |
| Body shape | **0.458***** | 0.013 | **0.038*** | -0.027 |

**Table C.** Correlations (*r*) between female or male participant traits and the strength of linear selection (β) on male figure traits for: a) attractiveness, and b) fighting ability for **paid online surveys**. Relative weight is participant weight controlled for height (i.e., equivalent to body mass index given isometry). Bold values indicate statistical significance. Asterisks indicate FDR rate for all paid data ****P* < 0.001, ***P* < 0.01, **P* < 0.05 (see Methods).

| **a) Attractiveness** | |  |  |
| --- | --- | --- | --- |
| *Female participant traits* | | | |
| Male trait | Age | Height | Relative weight |
| Penis size | -0.062 | -0.07 | 0.137 |
| Height | -0.07 | 0.078 | -0.165 |
| Body shape | 0.042 | -0.036 | 0.022 |
| *Male participant traits* | | | |
| Male trait | Age | Height | Relative weight |
| Penis size | **0.346**** | -0.111 | 0.078 |
| Height | -0.135 | 0.109 | -0.066 |
| Body shape | -0.095 | 0.18 | -0.167 |
| **b) Fighting ability** | |  |  |
| *Male participant traits* | | | |
| Male trait | Age | Height | Relative weight |
| Penis size | 0.060 | 0.086 | 0.093 |
| Height | -0.041 | -0.025 | -0.100 |
| Body shape | -0.07 | -0.2 | -0.22 |

**Table D.** Results from general linear mixed models with parameter estimates and chi-square (χ2) test statistics, including response time as the dependent variable and the three standardised male traits as fixed covariates for male attractiveness, and fighting ability in **paid online** surveys with either a response time threshold (≤20s) or all data. Bold values indicate statistical significance with original *P* values after accounting for a false discovery rate for multiple comparisons for all paid data (see Methods).

| ***Online paid (subset data)*** | |  |  | ***Online paid (all data)*** | |  |  |
| --- | --- | --- | --- | --- | --- | --- | --- |
| **a. Female rating of male attractiveness** | | | | | | | |
| Traits | Estimate | χ2 | *P (χ2)* | Traits | Estimate | χ2 | *P (χ2)* |
| (Intercept) | 8.384 |  |  | (Intercept) | 8.418 |  |  |
| Penis size | 0.043 | 52.578 | **<0.00001** | Penis size | 0.048 | 47.687 | **<0.00001** |
| Height | 0.003 | 0.292 | 0.589 | Height | 0.001 | 0.007 | 0.935 |
| Body shape | 0.037 | 38.101 | **<0.00001** | Body shape | 0.039 | 32.442 | **<0.00001** |
| **b. Male rating of rival’s attractiveness** | | | | | | | |
| Traits | Estimate | χ2 | *P (χ2)* | Traits | Estimate | χ2 | *P (χ2)* |
| (Intercept) | 8.119 |  |  | (Intercept) | 8.171 |  |  |
| Penis size | 0.028 | 17.498 | **0.00003** | Penis size | 0.025 | 8.524 | **0.004** |
| Height | 0.008 | 1.216 | 0.27 | Height | 0.006 | 0.515 | 0.473 |
| Body shape | 0.027 | 15.813 | **0.00007** | Body shape | 0.03 | 12.192 | **0.0005** |
| **c. Male rating of rival’s fight ability** | | | | | | | |
| Traits | Estimate | χ2 | *P (χ2)* | Traits | Estimate | χ2 | *P (χ2)* |
| (Intercept) | 8.046 |  |  | (Intercept) | 8.109 |  |  |
| Penis size | 0.018 | 5.959 | **0.015** | Penis size | 0.005 | 0.252 | 0.615 |
| Height | -0.001 | 0.019 | 0.89 | Height | -0.003 | 0.13 | 0.718 |
| Body shape | 0.026 | 12.229 | **0.0005** | Body shape | 0.015 | 2.33 | 0.127 |
