# Supplementary material for: Experimental evidence that penis size, height, and body shape influence assessment of male sexual attractiveness and fighting ability in humans
Source: PLoS Biol. 2026 Jan 22;24(1):e3003595. doi: 10.1371/journal.pbio.3003595 (PMC12826512; doi:10.1371/journal.pbio.3003595)
Supplement: Attachment — Submitted filename: Response to reviewers_24 Nov.docx [file pbio.3003595.s006.docx]

24 Nov 2025

RE: Decision on Manuscript ID PBIOLOGY-D-25-02783R1

Dear Dr Roberts,

Thank you for your constructive and supportive feedback on our manuscript (formerly "Effect of penis size, height and body shape on sexual attractiveness, perceived attractiveness and assessment of fighting ability in human"). We were pleased to receive such positive and insightful reviews from all three reviewers and are grateful for the opportunity to revise our manuscript.

We have addressed all the specific points raised by the editorial team and the reviewers. Below, we provide a point-by-point response to all comments, detailing the changes made. We have also uploaded a clean, revised manuscript and a 'track-changes' version.

We believe the manuscript has been significantly strengthened by these revisions, particularly by the suggestions from Reviewer 3 to re-frame our main findings, which we have incorporated into the Discussion.

We look forward to your assessment of the revised manuscript and hope it is now suitable for publication in *PLOS Biology*.

Sincerely,

The authors

**EDITOR'S COMMENTS TO THE AUTHORS:**

IMPORTANT - please attend to the following:

a) Please can you change the Title to include an active verb? We suggest: "The penis size, height and body shape of human males influence sexual attractiveness, perceived attractiveness and assessment of fighting ability"

**Response:** Done. As suggested, we have now changed the title to: “Experimental evidence that penis size, height and body shape influence assessment of male sexual attractiveness and fighting ability in humans”

b) Please attend to all of the requests from the reviewers.

**Response:** We have attended to all requests from the reviewers, as detailed in the point-by-point responses below.

c) Please ensure that you comply with our Data Policy; specifically, we need you to supply the numerical values underlying Figs 2ABCDEF, 3ABCDEF, 4ABCDEF, 5ABC, 6ABC, 7ABC, either as a supplementary data file or as a permanent DOI’d deposition. My understanding is that the data and code provided in S1 Data are sufficient to recreate the Figures; please can you confirm whether this is the case?

**Response:** We confirm that the data and code provided in S1 Data are sufficient to recreate all figures, including Figs 2ABCDEF, 3ABCDEF, 4ABCDEF, 5ABC, 6ABC, and 7ABC.

d) Please cite the location of the data clearly in all relevant main and supplementary Figure legends, e.g. “The data underlying this Figure can be found in S1 Data” or “The data underlying this Figure can be found in <https://zenodo.org/records/XXXXXXXX>

**Response:** Done. We have now added the sentence “The data underlying this Figure can be found in S1 Data” to the end of the legends for Figures 2 (Lines: 429-430), 3 (Lines: 436-437), 4 (Lines: 444-445), 5 (Lines: 452-453), 6 (Lines: 460-461), and 7 (Lines:468-469).

**REVIEWERS' COMMENTS:**

Reviewer #1:

In this manuscript the authors use over computer-generated images displayed life size in person or online to test how male genital size, height and body size influences assessment of a male's attractiveness by females, and males' perception of fighting ability and attractiveness to females. The design allows the authors to separately determine the affect of the three traits independently aa well as interactions between them. The main new result is that males use male genital size to assess male fighting ability and attractiveness to females. This suggests that male-male competition may have played a role in the evolution of the unusually large genitalia of human males.

I do not have any substantial criticism of this paper. The analyses are competent and thorough and the results are clear. I do think the authors could make it a little more clear why the female data is included here given that the results are those published some years ago in PNAS. They do not this, but I think it could be made much more clear in the discussion, including a statement of what it has added to the current analyses.

**Response**: We thank the Reviewer for their positive feedback on our manuscript. We have now clarified in both the methods and discussion sections that our current study includes data from the 2013 PNAS paper [35], which only looked at the in-person effects of male morphology on female assessment of sexual attractiveness. By adding these data, we can run statistical tests to directly compare the effect of penis size, height and body shape on female mate choice and male-male assessment for both in-person and online surveys using identical stimuli (and contrast the effects for male and female participants). The passages in the Methods and Discussion now read as follows:

“In total, we recruited over 600 men and over 200 women (approximately 100 for each of the 8 surveys). In addition, we incorporated data from a previous study [35] into our analysis, in which we ran an in-person assessment of male attractiveness by female participants (n = 105) using the same stimuli that we used in the current study. Inclusion of the data from the past study [35] provided us with the opportunity to directly compare the relative strength of intersexual selection (female choice) and intrasexual selection (male-male assessment) using identical stimuli for both in-person and online surveys.” Lines: 245-253

“By re-analysing the findings of [35] alongside our new data on male assessment of rival figures and the use of both in-person and online surveys of participants of both sexes, our study provides evidence that pre-copulatory sexual selection due to both female choice and male-male competition might have contributed to the enlarged size of the human penis when compared to that of other primates.” Lines: 583-587

I have marked "accept" in my recommendation. However, I have to note that I was unsurprised by the results and I do not think many in the field will be surprised. My view of this is that height, broad shoulders and genital size are all traits that distinguish males. That females prefer "male" traits and the same traits indicate competition in males is not particularly surprising to me, given our broader knowledge of sexual selection in animals.

**Response**: As biologists who mainly work on non-human animals, we can only note that our results were unexpectedly (or at least unexpectedly clear). There is very little evidence that male genital size affects female choice in other animals, and there is no robust evidence that male genital size is used to assess fighting ability. Our results were surprisingly clear-cut because one might expect any effect of penis size on assessment of fighting ability to be hard to detect, given that height and body shape are closely functionally linked to fighting ability, making them excellent cues of fighting ability, while this is not the case for penis size.

Though the results are unlikely, in my opinion, to surprise those in the field, the study is strong in methods and analyses, and it will surely attract substantial attention in the general science press.

**Response**: We thank the Reviewer for their positive recommendation on our manuscript. Our motivation for the study was to provide the *first robust experimental test* of a key, long-standing hypothesis that had previously lacked direct support. So, while our results may not be surprising to those in the field, claims about the role of penis size in the assessment of fighting ability had not been empirically demonstrated before. By experimentally separating penis size from confounding covariation with traits like height and body shape, our study provides the first direct evidence that penis size affects the assessment of fighting ability by rivals. We are pleased that the reviewer finds this evidence convincing, and we hope this empirical validation of a major hypothesis is a valuable contribution to the field.

Reviewer #2:

[identifies himself as Bogusław Pawłowski]

This is very interesting paper with quite rigid methods used by the authors. The results are very interesting in a few aspects. Firstly, when controlling for other studied traits they found that a man's height, body shape and penis size are related to perceived attractiveness and assessment of fighting men ability by other males. It supports the hypothesis on the sexual selection for these three traits. Secondly, they found that the way the study is conducted (online vs in person) may affect some results. Might be it is rather a matter of showing stimuli in life sizes (in the in-person study), which method looks as more relevant for such studies (what the authors also underline).  Thirdly, as could be expected it is body height and body shape that are more important both for attractiveness and the assessment of fighting ability, than penis size itself. Fourthly, they showed sex differences in the sexual selection pressure which appeared to be a bit stronger in the case of male intrasexual selection ("…there was no evidence for diminishing effects (as it was in the case of women assessing attractiveness) of increased penis size, being taller or a greater shoulder-to-hip ratio on male assessment of other males' attractiveness to females"). Fifth, they showed that assessments might depend on a subject's own body morphology (or in one case a male subject age). What is also interesting, is that response time was related to the perceived stimuli traits. Might be the author could then mention in the Discussion that the perception in the studied aspects is "better tuned" to exclude some traits (this aligns better with the "bad genes hypothesis" than with "good genes hypothesis") than to choose the best one from the "good ones".

As far as attractiveness assessments by women is concerned, it seems to by rather replication of the study on exactly the same stimuli published in 2013 (<https://doi.org/10.1073/pnas.1219361110>). I think it should be then clearly written in the paper that this part of the present study in fact replicates the previous study.  The original part of the present study is related to both males' assessments (the level of sexual competition "jealous" and fighting ability).

**Response:** Done. As detailed in response to a similar comment from Reviewer 1, we have now clarified the text regarding the reanalyses of data from the earlier PNAS paper. In sum 88% of the data reported in the current study is new (approximately 800 of 900 participants). The revised passage reads as follows:

“In total, we recruited over 600 men and over 200 women (approximately 100 for each of the 8 surveys). In addition, we incorporated data from a previous study [35] into our analysis, in which we ran an in-person assessment of male attractiveness by female participants (n = 105) using the same stimuli that we used in the current study. Inclusion of the data from the past study [35] provided us with the opportunity to directly compare the relative strength of intersexual selection (female choice) and intrasexual selection (male-male assessment) using identical stimuli for both in-person and online surveys.” Lines: 245-253

The stimuli used by the authors have flaccid penises and since it has been already shown that flaccid penis length is positively related to the penis size in erection, the authors should refer to the adequate papers (some references are needed). It is because that it is the erect penis (as in some other primates) that can be used to deter e.g. males from other group (and not the flaccid one). The question is then if the stimuli with the erect penises could reveal stronger effect than the stimuli with the flaccid penises.

**Response:** Done. We have now revised our discussion to include the information highlighted by the reviewer. The revised text now reads, as follows:

“It is important to note that our computer-generated figures had flaccid penises. Flaccid penis length is known to be positively correlated with erect length [59]. While a flaccid penis is a more common state in humans and probably a more relevant signal available to assess a rival’s fighting ability, male-male displays in other primates and female choice in humans can involve erect penises [1, 2]. It is plausible, therefore, that using figures with an erect penis might reveal an even stronger effect of penis size on sexual attractiveness and assessment of fighting ability. Future studies using erect penises remain an avenue for future research to test this claim.” Lines: 748-760

The discussion of the results and the difference between online and in-person results is convincing. To make height more relevant stimulus in online study, I would suggest rather showing pairs (having the chance of comparison in one picture) in which e.g. the shorter silhouette would be the imaginable size of a subject which is supposed to assess a rival attractiveness or fighting ability. This is a method used when studying preference of sexual dimorphism in height between partners, and in the case of the present study it can be converted e.g. to two men silhouettes. The result that taller women pay more attention for a man height, confirms previous studies (taller male norm in WEIRD countries).

**Response**: The reviewer raises an excellent suggestion for future studies. For the current research, however, we wanted to keep the online and in-person experimental designs as similar as possible (i.e., single-stimulus presentation) to allow for a direct and cleaner comparison between the delivery modes. It should also be noted that there was a training period immediately before data collection. During this training period, participants were exposed to 13 figures that spanned the height range and therefore had the opportunity to notice that the height of the figures varied.

Minor points:

There is 73 = 343  and should be 73 = 343

**Response**: This has been corrected. We now use the superscript format: 7^3^ = 343. Lines: 143-144

Does in online study the subjects also saw each time only one picture  (gif) at a time as in the in-person study - it should be clarified.

**Response:** Yes, they did. We have clarified this in the Methods. The sentence now reads:

" As with the in-person surveys, participants were randomly assigned one of the 100 unique sets of figures. They completed a questionnaire before sequentially viewing the 13 training figures and then viewed the 56 figures in the test set, with each figure presented one at a time. This procedure is identical to the procedure for in-person surveys." Lines: 229-232

On p. 15 it is "males rating male attractiveness" - which seems not be true according to the previous information ("competitiveness")

**Response**: We have now revised the sentence in the Methods (former p.15) to clarify the content, as follows:

“In both cases, there were three types of surveys: females rating male attractiveness, males rating a rival male’s attractiveness, and males rating a rival male’s fighting ability.” Lines: 195-196

We explain and justify ‘a rival's attractiveness’ as a proxy for sexual competition further in the following paragraph, where we write:

“To determine how male participants estimated the attractiveness of these figures to females, they were asked to rate figures based on the question: 'How jealous would you feel if you saw this man talking to your partner?’ We assume that increased jealousy is related to a rival being perceived to be more sexually competitive.” Lines: 211-214.

We therefore interpreted this jealousy rating as a proxy for the 'rival's attractiveness to females (i.e., their sexual competitiveness)’.

We then use this consistent terminology ‘Male rating of a rival’s attractiveness’ throughout the manuscript, including all relevant sub-headings in the Figures and Results, such as:

***Female rating of male attractiveness***, Line: 345

***Male rating of a rival’s attractiveness***, Line: 368

***Male rating of a rival’s fighting Ability***, Line: 390

We hope this revision, combined with the explicit justification in the Methods, now makes our interpretation clear.

The authors write: "We used the unpaid surveys to reduce the risk that some participants did not pay attention to the figures and only completed the survey for financial gain. T".   So, what is the sense to collect the data paying for participation?

There are some tables in Suppl. Mater., but it would be worth to discuss the difference between paid and unpaid (might it is not a problem of being (or not) paid but the demographic (e.g. age) structure is different in these two groups.

**Response**: Our rationale for collecting both paid and unpaid data was twofold: 1) to increase our overall sample size, and 2) to check the robustness of the findings across different recruitment modes.

The reviewer’s comment made us realise that our original wording in this section, which explains the difference between online paid and unpaid data (very similar vs. significant effect), may have been unclear. To clarify:

1. The 'significant effect of payment' (**Table S2**) referred only to a difference in the mean scores (i.e., the statistical intercept) between the paid and unpaid data.
2. The key scientific findings—the patterns of selection (i.e., the slopes for how each trait affected ratings)—were qualitatively very similar for the two groups (as shown in Appendix **A1-4**).

While we did not run a formal comparison, the fact that the selection gradients themselves were congruent gave us confidence in our findings. We therefore chose only to present the unpaid sample in the main text as a precaution against any risk of inattentive, financially motivated responses. We have substantially revised this paragraph in the Methods to make our rationale for only presenting unpaid data clearer. The revised paragraph now reads, as follows:

“We compared the paid and unpaid online surveys and found that there was a significant effect of payment on the mean scores (i.e., the intercept; **Table S2**). Crucially, however, the patterns of selection (i.e., the slopes for how each male trait affected a figure's rating) were qualitatively very similar for the two groups (see Appendix **A1-4**). Given the consistency in these key findings, we chose to present only the unpaid sample in the main text. This simplifies the analysis and reduces any potential risk that some of the paid participants were less attentive to the survey figures or completed the survey only for financial gain.” Lines: 259-265

Due to the results obtained (selection was shown to be usually stronger on height or body shape than on penis size) I would suggest to change the title into for instance:
a.)     Body height, shape and penis size - perceived attractiveness and assessment of fighting men ability, or

b.)     The effect of height, body shape and penis size on perceived attractiveness and assessment of fighting ability in men.

**Response**: We thank the reviewer for the above suggestions. The Editor also made a suggestion for amending the title, and we have decided to use similar wording to their recommendation.

I am not sure if Tab S4 is necessary. The results presented in this tab. are similar to Tab. 3

**Response**: The reviewer is correct that the results are very similar. **Table 3** shows the analysis with response times >20s removed, while Table **S4** shows the analysis with all response times. We included **S4** to demonstrate that our data trimming decision did not bias the results. We feel it is valuable for transparency, but we are happy to remove it if the Editor feels it is unnecessary. For now, we have left it in the supplementary material.

Reviewer #3:

I enjoyed reading this paper. It covers a topic that will be of broad interest, and the methods, analysis and writing are good. An advantage is the investigation of linear, quadratic, and correlational selection gradients, which is somewhat uncommon in human sexual selection research. Another advantage is the collection of both in-person/life-size data and online data. I have the following suggestions for strengthening the manuscript, particularly in regard to theoretical framing and interpreting the results:

1.  Sexual ornament vs. status badge: Effects of flaccid penis length on perceptions of both attractiveness and fighting ability are interesting and novel. The authors state they "believe that the most important finding of our study is that penis size influences how males assess a rival's fighting ability." I agree that this is interesting, but to me, the most interesting finding is that the effect of penis size on attractiveness to women is approximately 5 times the magnitude of the effect on male perceptions of fighting ability. One reason that this finding is interesting and important is that it is the reverse relationship from other putative display traits in human males, such as beards (Neave & Shields, 2008) and deep voices (Aung et al., 2024), which more strongly influence perceptions of dominance and fighting ability among males than they increase attractiveness to females. In other words, whereas beards and deep voices appear to function primarily as badges of status (Aung et al., 2023; Grueter, Isler, & Dixson, 2015), penile length looks like a sexual ornament from the present data. A thorough review of sexual selection in human males that provides evidence that male secondary sex traits function primarily in contests is Puts, Carrier, and Rogers (2022). The present results suggest that penis size is an important exception to this overall pattern.

**Response:** We thank Prof Puts for this insightful suggestion. We have substantially revised the Discussion to incorporate this "ornament vs. status badge" framework, as suggested. The revised paragraph with recommended references reads, as follows:

“It should, however, be noted that selection on penis size, because it elevates perceived fighting ability, was much weaker than selection on height or body shape (three- to eight-fold less; **Table 1**). Assessment of the strength of a rival is therefore mainly determined by his height and body shape. Likewise, the effect of penis size on male-perceived fighting ability was much weaker (four- to seven-fold less; **Table 1**) than its effect on attractiveness to females. This finding is intriguing as opposite patterns have been reported for other male secondary sexual traits, such as beards [61] and deep voices [62], which more strongly influence male perceptions of dominance and fighting ability than attractiveness to females. This contrast suggests that while many sexually dimorphic male traits function primarily as 'badges of status' for male-male contests in humans [63-65], penis size might function primarily as a 'sexual ornament' for attracting mates. This aligns with our findings that traits like height and body shape explains evaluation of a rival’s fighting ability, whereas penis size seems to have a stronger effect as a display trait that affects female assessment of attractiveness. Indeed, while body shape was the strongest driver of male attractiveness to females, penis size and height had very similar effects on male attractiveness for the in-person survey, and penis size actually had a far stronger effect than height for the online survey. While our study confirms the role of penis size in assessment of a rival’s fighting ability, the main source of sexual selection for enlargement seems to be female choice.” Lines: 678-696

In my 2010 EHB paper that the authors reference, I asked: "Are any of men's traits properly considered sexual ornaments (i.e., function primarily in mate attraction)? Men's penises are longer and thicker, both relatively and absolutely, than those of our closest relatives, chimpanzees and gorillas, and could have evolved to signal mate quality. Women report greater satisfaction with larger penises (Lever, Frederick, & Peplau, 2006), so penis size may affect a man's ability to stimulate orgasm in women (Miller, 2000). Female orgasm may boost sperm retention, facilitate sperm activation, and encourage additional copulations (reviewed in Puts & Dawood, 2006, see also Gallup et al., 2003). However, it has also been suggested that penis size may advertise vigor to other men (Diamond, 1997)." The present results thus provide the strongest evidence yet addressing this question. On this theme, I appreciated the authors' discussion of ancestral constraints on female mate choice, which is probably accurate; however, they might also note that the existence of strong female preferences in modern humans suggests that these preferences were functional ancestrally.

**Response:** We thank the reviewer for their additional insights. We have now added the idea to the conclusion section that female preferences for penis size in modern humans suggest that these preferences were ancestrally functional. We have further noted the role of penis size in female orgasm and its subsequent role in copulation. The revised passage, which now includes the suggested references, reads as follows:

“The existence of the strong female preference for greater penis size that we observed in our study suggests that these preferences are likely to have been present in our ancestors, and therefore will have had some role in sexual selection, causing the evolution of the enlarged human penis. It is also worth noting that penis size and shape may have been selected for because of their effects on sexual stimulation, specifically by increasing the likelihood of female orgasm, which has, in turn, been linked to improved sperm retention, facilitating sperm activation, and encouraging additional copulations [6, 76-77]. It therefore seems likely that both pre- and post-copulatory sexual selection have contributed to the evolution of human penis size.” Lines: 779-789

2. Primary functions in display vs. fighting success: It seems important to distinguish between traits that function more directly in winning fights, such as size and muscularity, and those that seem to function more in display, such as deep voices, and perhaps penis length. In the context of the present paper, it makes sense that males and females would have evolved sensitivity to the size and body shape of males because of what those traits indicate about a male's fighting ability, genetic quality, status, etc., but greater male size and strength evolved tens of millions of years ago, probably mainly in the service of winning fights directly, and perceptions evolved secondarily to that. This is different from penis size.

**Response:** We agree that this is an important distinction and have modified our discussion to explicitly state the stronger role that penis size plays in display in comparison to male fighting ability. In the ‘Penis size and assessment of fighting ability’ subsection of Discussion, we now write:

“It should, however, be noted that selection on penis size, because it elevates perceived fighting ability, was much weaker than selection on height or body shape (three- to eight-fold less; **Table 1**). Assessment of the strength of a rival is therefore mainly determined by his height and body shape. Likewise, the effect of penis size on male-perceived fighting ability was much weaker (four- to seven-fold less; **Table 1**) than its effect on attractiveness to females. This finding is intriguing as opposite patterns have been reported for other male secondary sexual traits, such as beards [61] and deep voices [62], which more strongly influence male perceptions of dominance and fighting ability than attractiveness to females. This contrast suggests that while many sexually dimorphic male traits function primarily as 'badges of status' for male-male contests in humans [63-65], penis size might function primarily as a 'sexual ornament' for attracting mates. This aligns with our findings that traits like height and body shape explains evaluation of a rival’s fighting ability, whereas penis size seems to have a stronger effect as a display trait that affects female assessment of attractiveness. Indeed, while body shape was the strongest driver of male attractiveness to females, penis size and height had very similar effects on male attractiveness for the in-person survey, and penis size actually had a far stronger effect than height for the online survey. While our study confirms the role of penis size in assessment of a rival’s fighting ability, the main source of sexual selection for enlargement seems to be female choice.” Lines: 678-696

3. Primary function of penis size in display vs. sexual stimulation: The authors discuss copulatory sexual selection on male genitalia in their introduction and then note that precopulatory sexual selection on male genitalia is understudied; hence, the focus of the present paper. It might be worth returning in the discussion to the idea that both forms of sexual selection may have been important pressures on human penile length. Related to this and the idea that orgasm increases the probability of fertilization mentioned in the introduction, a thorough review of the evidence is Wheatley and Puts (2015).

**Response**: As mentioned above, in the conclusion section, we now discuss the role of penis size in female orgasm and its subsequent role in copulation and fertilisation success to flag the idea that both pre- and post-copulatory sexual selection might have played an important role in the evolution of human penis size. The revised passage, including the recommended references, reads as follows:

“The existence of the strong female preference for greater penis size that we observed in our study suggests that these preferences are likely to have been present in our ancestors, and therefore will have had some role in sexual selection, causing the evolution of the enlarged human penis. It is also worth noting that penis size and shape may have been selected for because of their effects on sexual stimulation, specifically by increasing the likelihood of female orgasm, which has, in turn, been linked to improved sperm retention, facilitating sperm activation, and encouraging additional copulations [6, 76-77]. It therefore seems likely that both pre- and post-copulatory sexual selection have contributed to the evolution of human penis size.” Lines: 779-789

4. Why does penis length influence perceptions of fighting ability? "One possible explanation why a larger penis might be an honest signal of fighting ability is that testosterone influences penile development at puberty, and higher levels of testosterone in adult males are associated with increased muscle mass, greater aggression, and higher competitive ability [42, 57-58." Agreed. Another reason may be that flaccid penis length responds to stress/anxiety by shortening as adrenaline redirects blood flow away from the penis. This response may function to decrease injury to the penis during fight or flight situations. Research subjects may be interpreting penis length partly as a state rather than a trait, with longer penises perceived as indicating confidence, status, and a relative absence of plausible threats.

**Response**: We have revised this section of our Discussion to include this second hypothesis. The added passage reads, as follows:

“A second, non-mutually exclusive explanation is that flaccid penis length functions as an indicator of a male's physiological state. Flaccid penis length can shorten in response to stress or anxiety as adrenaline redirects blood flow away from the genitals, a response that may function to reduce injury during 'fight or flight' situations [59-60]. Therefore, participants may interpret a flaccid penis that is longer as a signal of rival confidence, lower stress, or the relative absence of perceived threat.” Lines: 671-676

5. Effects of perceivers' traits on their perceptions: The present finding of effects of own height on men's perceptions should be discussed in the context of a prior literature on this: (Watkins, Fraccaro, et al., 2010; Watkins, Jones, & DeBruine, 2010; Wolff & Puts, 2010). Watkins et al. found that less dominant and shorter men were more attentive to indicators of the dominance of other men, whereas Wolff & Puts found no relationship. I believe I've seen one or two other studies on this topic more recently as well. The finding of an effect of male perceiver's age on perceptions male body shape is similar to the finding of Aung et al. (2024) that low voice pitch more strongly influenced perception of fighting ability in younger men and perceptions of social status in older men. Like the present authors, Aung et al. interpreted their results in the context of age-related changes in the ways that male compete.

**Response**: We thank Prof Puts for these suggestions and for providing the specific literature to contextualise our findings. We have now revised the ‘Participants' own traits’ subsection of the Discussion to incorporate these points and citations. The updated paragraph reads, as follows:

“For male participants, the only relationship observed for both the in-person and online surveys is that a more V-shaped body of a rival significantly more strongly influenced younger than older males when evaluating a rival’s attractiveness. This finding is in line with those of [62], who found low voice pitch (another dominance-related trait) more strongly influenced perceptions of fighting ability in younger than older men. One explanation for such results is that younger males are more likely to engage in physical fights and therefore assess traits associated with strength and dominance, while older males may rely more on social status or coalitions to prevent fights occurring, and instead assess traits linked to reproductive success when they evaluate rivals [68]. For the in-person surveys, penis size significantly and more strongly influenced older than younger males when assessing the attractiveness of a rival. This suggests that perceptions of sexual competition might increase with age, perhaps driven by experience gained in how females evaluate male attractiveness. There was, however, no equivalent relationship for the online survey. Finally, taller males were significantly more attentive to height when evaluating a rival’s attractiveness. This result adds to the existing literature exploring how a man's own dominance affects his perception of rivals. The results of studies have been mixed. For example, some studies have found that shorter or less dominant men are more sensitive to dominance cues in rivals [69], whereas others found no such detectable relationship [e.g., 70]. One explanation is that shorter men do not need to distinguish between slightly taller and much taller than average males because they are both more attractive than a short man. Again, however, this pattern was apparent for our in-person survey but not for our online survey.” Lines: 709-731

6. Typos/minor edits:  "In humans, a few experimental studies have presented women with computer generate images…" Change to "generated"    "…600 male and 200 female participants ranked figures that independently varied in three traits" Change to rated?

**Response**: Done. See lines: 120; 142

I hope these comments help interpret and explain the importance of their study. These are unique data that shed significant light on major questions in human sexual selection.

- Signed, David Puts

**Response**: Thank you for the feedback and for providing these key citations. We have now contextualised our findings as requested in the Discussion section, and we hope the reviewer finds these changes satisfactory.

References

Aung, T., Hill, A. K., Hlay, J. K., Hess, C., Hess, M., Johnson, J., . . . Puts, D. (2024). Effects of Voice Pitch on Social Perceptions Vary With Relational Mobility and Homicide Rate. Psychological Science, 35(3), 250-262. doi:10.1177/09567976231222288

Aung, T., Hill, A. K., Pfefferle, D., McLester, E., Fuller, J., Lawrence, J. M., . . . Puts, D. A. (2023). Group size and mating system predict sex differences in vocal fundamental frequency in anthropoid primates. Nature communications, 14(1), 4069. doi:10.1038/s41467-023-39535-w

Grueter, C. C., Isler, K., & Dixson, B. J. (2015). Are badges of status adaptive in large complex primate groups? Evolution and Human Behavior, 36(5), 398-406.

Neave, N., & Shields, K. (2008). The effects of facial hair manipulation on female perceptions of attractiveness, masculinity, and dominance in male faces. Personality and Individual Differences, 45(5), 373-377. doi:10.1016/j.paid.2008.05.007

Puts, D., Carrier, D., & Rogers, A. R. (2022). Contest competition for mates and the evolution of human males. In D. M. Buss (Ed.), The Oxford handbook of human mating (pp. 317-377). Oxford, UK: Oxford University Press.

Watkins, C. D., Fraccaro, P. J., Smith, F. G., Vukovic, J., Feinberg, D. R., DeBruine, L. M., &

Jones, B. C. (2010). Taller men are less sensitive to cues of dominance in other men. Behavioral Ecology, 21(5), 943-947. doi:10.1093/beheco/arq091

Watkins, C. D., Jones, B. C., & DeBruine, L. M. (2010). Individual differences in dominance perception: Dominant men are less sensitive to facial cues of male dominance. Personality and Individual Differences, 49(8), 967-971. doi:10.1016/j.paid.2010.08.006

Wheatley, J. R., & Puts, D. A. (2015). Evolutionary science of female orgasm. In T. K. Shackelford & R. D. Hansen (Eds.), The Evolution of Sexuality (pp. 123-148): Springer.

Wolff, S. E., & Puts, D. A. (2010). Vocal masculinity is a robust dominance signal in men. Behavioral Ecology and Sociobiology, 64(10), 1673-1683. doi:10.1007/s00265-010-0981-5
